# Primary eye care in Nepal: current situation and recommendations for integration

**Published:** 2022-03-01

**Authors:** Reeta Gurung, Radhika Upreti Oli

**Affiliations:** 1 Chief Executive Officer: Tilganga Institute of Ophthalmology, Kathmandu, Nepal.; 2 Senior Research Officer: Tilganga Institute of Ophthalmology, Kathmandu, Nepal.

**Trained primary health care workers and mid-level ophthalmic professionals at the existing primary health care networks are needed to provide crucial first-level ophthalmic care.**

## Overall progress in eye care

In the last four decades in Nepal, following the landmark national blindness survey in 1981, there has been significant progress in the field of eye care. The estimated number of people with blindness decreased from 118,000 in 1981 to 93,000 in 2012 despite the growth in population. The number of ophthalmologists increased from seven in the 1980s to 400 in 2020. The eye care infrastructure, including community eye centres, eye departments, and eye hospitals, increased from five in 1981 to more than 100 in 2010. These improvements led to a reduction in the prevalence of national blindness from 0.84% in 1981 to 0.35% in 2010.^1^^,^^2^

Nepal was one of the first countries in South Asia to implement VISION 2020: The Right to Sight. It adopted disease-focused strategies and vertical eye health programmes to tackle common diseases causing blindness.^3^ By 2018, Nepal successfully eliminated trachoma as a major public health concern, thanks to the National Trachoma Programme, a public–private partnership launched in 2002.^4^

Nepal is a signatory to the World Health Organization's global eye health action plan 2014–2019. The country's National Health Policy 2019 has provided for the development and expansion of eye care services through public–private partnerships in all three tiers of government: federal, provincial, and local; the integration of primary eye care with primary health care; and the coordination of eye care programmes by a dedicated eye unit at the federal ministry of health.^5^ Provincial governments have started the ‘one school, one nurse’ programme to provide basic health care, including eye care to school-going children.

Nepal's public health insurance scheme was launched in 2016–17, now covering 75 out of 77 districts. The main objective of the scheme is to increase the financial protection of the public by promoting pre-payment and risk pooling in the health sector. Any Nepalese family paying the premium amount of 3,500 NPR (about USD 29.5) per annum set by the Health Insurance Board can get the benefits of the package irrespective of their employment status. The benefits cover outpatient eye care, emergency hospital care, ophthalmic investigation, minor and major surgeries, and ocular medicines. These services can be availed of in public and private hospitals recognised by the Health Insurance Board of Nepal.^6^

Despite the developments in eye health care, the incidence of blindness and vision impairment has not reduced as expected. The reasons are population growth, ageing, inequitable distribution of resources, and lack of integration between levels of eye health care. The pattern of disease has changed from acute infections to chronic ones.^1^^,^^2^ The 2011 mid-term review of the VISION 2020 programme revealed that eye care services were not integrated with primary health care; nor was the modality of partnership with non-governmental organisations (NGOs) and the private sector well defined. Most of the NGO-run eye care programmes were struggling financially, and adequate attention was not given to universal health access.^7^ The low enrolment and high dropout rates in the national health insurance scheme have meant poor utilisation of the available eye care benefit packages.^8^ As a result, patients mostly end up having to pay for cataract operations.

## NGO-run primary eye care services

Primary eye care centres/community eye centres, usually run by NGOs, are fixed facilities where an ophthalmic assistant provides services such as management of common ocular disorders, health education, vision assessment, refraction, optical dispensing, school screening as part of outreach activities, and referral help. Most of these centres are located in district headquarters, which tend to be remote from villages in this hilly country. Consequently, more than 40% of the population is without basic eye services. Inevitably, people have to pay out of pocket, even for primary eye care.

About two years ago, NGOs partnered with municipal authorities to establish rural and urban eye clinics beyond district headquarters. In this model, the NGOs provide the equipment and technical support, while salaries and other running costs are borne by the municipality.

## Limitations and recommendations

Nepal's strong primary health care network covers all 77 districts and local *palikas*. The primary health care centres are run by primary health care workers, who often lack formal training in eye care. Lack of trained personnel is a big limitation of the primary health care programmes with respect to eye care.

At the community level, there is a great potential for training primary health care workers and female community health volunteers to promote eye health through early case detection and referral advice. This can help in the continued control of trachoma, prevention of corneal blindness, and control of eye diseases due to nutritional deficiency. The country needs more mid-level ophthalmic professionals, or ophthalmic assistants, to serve the need for increasing primary eye care services. Along with scaling up the existing community eye centres, trained mid-level ophthalmic professionals can be placed at primary health care centres to integrate eye care with general health care.

At the secondary level, district and zonal hospitals are already starting to set up eye departments run by ophthalmologists, where cataract services are available in a phased manner. We recommend that eye care departments with all the sub-speciality services should be set up at the province-level hospitals. They should be responsible for managing referral cases. In the difficult terrain of Nepal, telemedicine can be used to reach remote and as-yet-unreached sections of the population. The existing tertiary centres should be equipped with teaching, training, and research facilities.

## Conclusion

NGOs and the private sector have mostly driven eye care delivery in Nepal. The rural population continues to be deprived of basic eye care services, as primary health care workers are not trained to provide eye care. Eye care services by NGOs and the private sector are either not easily accessible or are expensive. The solution lies in training primary health care workers sufficiently so that they can provide the crucial first level of ophthalmic care. It is also necessary to increase the number of available trained mid-level ophthalmic professionals and depute them to government primary health care centres.

At the same time, the public-private partnership strategy, which has been effective in eliminating trachoma from Nepal, is a model which could be applied to the provision of essential eye care services. At all three levels of eye health service, the government should be the ultimate regulatory body.
